# Bromopride stimulates 5-HT_4_-serotonin receptors in the human atrium

**DOI:** 10.1007/s00210-025-04013-1

**Published:** 2025-03-17

**Authors:** Lina Maria Rayo Abella, Joachim Neumann, Britt Hofmann, Ulrich Gergs

**Affiliations:** 1 Institute for Pharmacology and Toxicology, Medical Faculty, Martin Luther University Halle-Wittenberg, Magdeburger Straße 4, D-06112 Halle (Saale), Germany; 2 Department of Cardiac Surgery, mid-German Heart Centre, University Hospital Halle, Halle (Saale), Germany

**Keywords:** Bromopride, Human atrium, Mouse atrium

## Abstract

Bromopride, an analogue of metoclopramide, is approved in some countries to treat gastrointestinal diseases. These therapeutic effects of bromopride are explained by antagonism at D_2_-dopamine receptors in the gut and the brain. We tested here the hypothesis that bromopride acts as an agonist or antagonist at the human cardiac 5-HT_4_-serotonin receptors. To this end, the force of contraction (FOC) was measured in isolated human atrial preparations (HAP), in isolated left atrial preparations (LA), and in isolated spontaneously beating right atrial (RA) preparations from mice with cardiac specific overexpression of the human 5-HT_4_-serotonin receptors (5-HT_4_-TG). Bromopride concentration dependently increased FOC in LA from 5-HT_4_-TG. The positive inotropic effect (PIE) of bromopride in LA from 5-HT_4_-TG was abolished by GR125487, a 5-HT_4_-serotonin receptor antagonist. Only in the presence of the phosphodiesterase III inhibitor cilostamide did bromopride raise FOC under isometric conditions in HAP. The PIE of 10 µM bromopride in HAP was extinguished by 1 µM GR125487. When serotonin had elevated FOC in HAP, additionally applied bromopride reduced FOC. These data suggest that bromopride is a partial agonist at human cardiac 5-HT_4_-serotonin receptors.

## Introduction

**Fig. 1:**
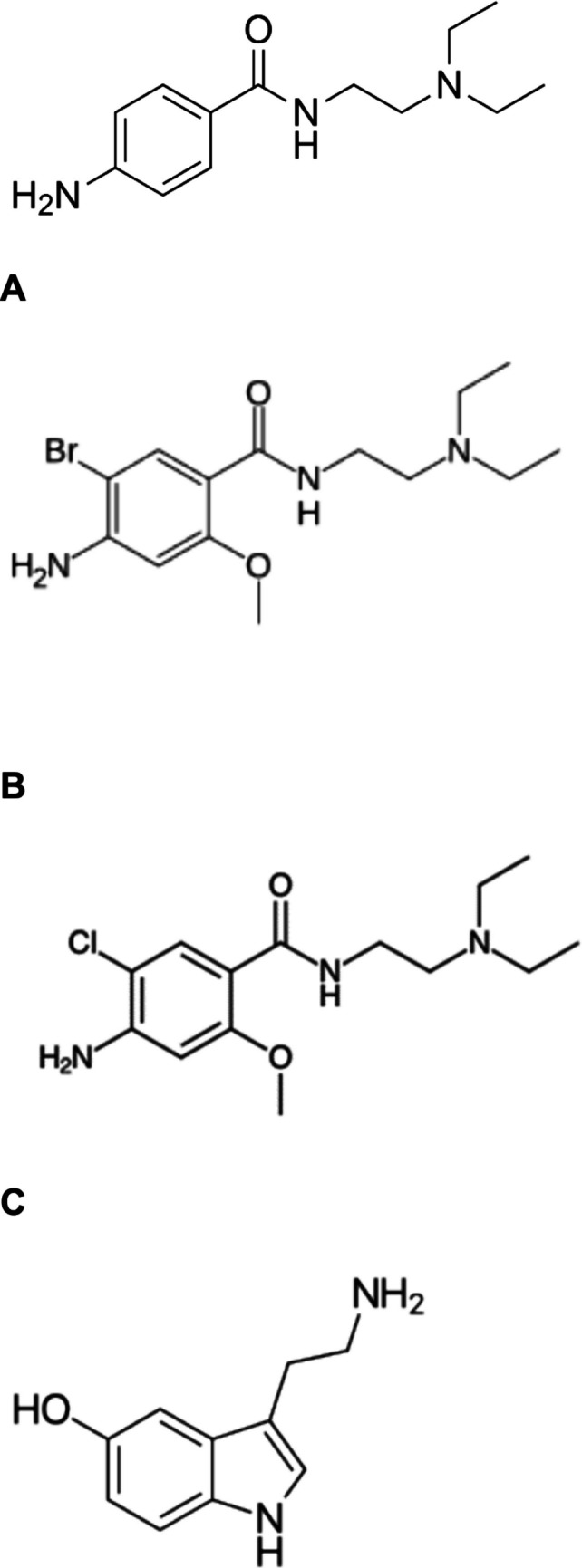
Comparison of structural formulae of procainamide (**A**), bromopride (**B**), metoclopramide (**C**), and serotonin (**D**). Comparison of the structural formulae of the mother compound procainamide with its derivatives metoclopramide and bromopride compared to the physiological agonist at serotonin receptors namely serotonin

Bromopride (2-methoxy-4-amino-5-**bromo**-N,N-diethylaminoethylbenzamide, Fig. 1A) presents itself as a structural analogue (containing in the benzene ring a bromine instead of a chlorine) of metoclopramide (2-methoxy-4-amino-5-**chloro**-N,N-diethylaminoethylbenzamide, Fig. 1A). Bromopride like metoclopramide can be viewed as a salicylamide or more generally as a benzamide like its congener procainamide (Fig. 1A).

As may be suspected from the nearly identical structural formulae (Fig. 1A), bromopride was initially developed together with other compounds like metoclopramide in search for analogues of the antiarrhythmic drug procainamide (Fig. 1A). But bromopride turned out to be too ineffective as an antiarrhythmic agent in preclinical studies (Mouille and Cheymol 1977). Therefore, other indications for bromopride were searched for in the gastrointestinal tract. Basically, bromopride has the same indications as metoclopramide. For instance, one uses peroral bromopride as a prokinetic drug in the gastrointestinal tract of patients (Tonini et al. 2004, Lachi -Silva et al. 2020, children: Epifanio et al. 2018, Fig. 2). Intravenous bromopride can stop emesis in patients receiving emetogenic chemotherapeutic drugs (Roila et al. 1985; Dunne et al. 2013). In contrast to metoclopramide, bromopride is approved in fewer countries, mainly in Southern Europe and South America (Dunne et al. 2013; Lachi-Silva et al. 2020).

**Fig. 2:**
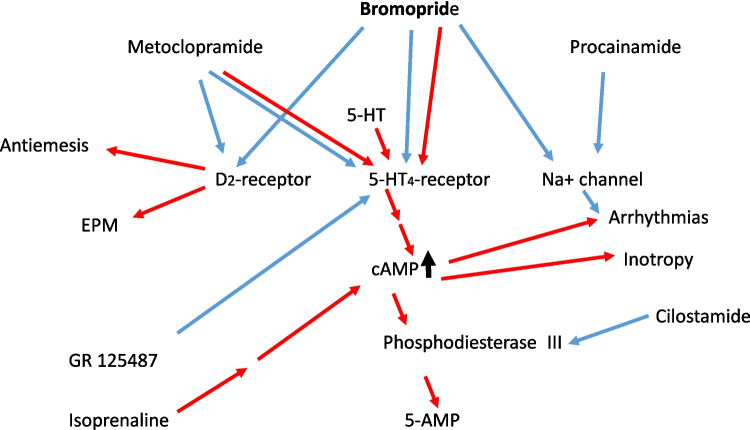
Mechanism(s) of action of bromopride in cardiomyocytes. Bromopride is compared here to other compounds and putative mechanisms of action of bromopride are indicated. Serotonin (5-HT) is an agonist (red arrow) at 5-HT_4_ receptors. Procainamide is an antagonist at cardiac sodium channels, typical of class I antiarrhythmic agents. In part, bromopride acts like procainamide. Stimulation of 5-HT_4_ receptors increases (black arrow) in the cardiomyocyte finally cAMP levels (Dolce et al. 2021). This cAMP not only can have a PIE but also can lead to arrhythmias (Kaumann and Sanders 1994; Dolce et al. 2021). The cAMP is degraded in human hearts mainly by phosphodiesterase III (inhibited by cilostamide) to inactive 5´-AMP. Isoprenaline increases via β-adrenoceptors (not shown but indicated by two red arrows) cAMP in the heart. GR 125487 is an antagonist at 5-HT_4_ receptors. Bromopride is an antagonist at dopamine D_2_ receptors in the brain and this explains not only the antiemetic but also Parkinson-like untoward effects (EPM) of bromopride. Metoclopramide is an agonist but also an antagonist at 5-HT_4_ receptors in the heart and an antagonist at dopamine D_2_ receptors in the brain. Stimulation of 5-HT_4_ receptors and inhibition of dopamine D_2_-receptors in the gut are thought to explain the beneficial intestinal effects of bromopride

Current thinking is that the beneficial gastrointestinal therapeutic effects of bromopride result by antagonism at central and intestinal D_2_-dopamine receptors with an IC_50_-value of about 2.1 µM, which is astonishingly large (Meltzer et al. 1983). Bromopride acts also in this regard like its sister compound metoclopramide. Both drugs exert Parkinson-like side effects that can be plausibly explained by an antagonism at the brain D_2_-dopamine receptor in patients (Tonini et al. 2004, Fig. 2).

We were motivated to study bromopride in more detail because we had investigated in the past its sister compound metoclopramide in the human heart (Neumann et al. 2021a). We reported metoclopramide to act as an agonist at 5-HT_4_-serotonin receptors in 5-HT_4_-TG and in isolated HAP (Neumann et al. 2021a). In addition, others, in a review suggested, that bromopride, based on its similarity to metoclopramide and other benzamides, might activate human 5-HT_4_-serotonin receptors in the gut (Tonini et al. 2004). It might be clinically relevant that bromopride acts as an agonist on cardiac 5-HT_4_-serotonin receptors because 5-HT_4_-serotonin receptors are present and functional in the human heart (Kaumann and Levy 2006). Thus, we were interested in the present study to ascertain (or refute) that bromopride might have cardiac side effects via 5-HT_4_-serotonin receptors. 5-HT_4_-serotonin receptors are the sole serotonin receptors to mediate the PIE and the positive chronotropic effects of serotonin in the human heart (Kaumann and Levy 2006). In order to obtain a small animal model for the study of human cardiac 5-HT_4_-serotonin receptors, we have generated and characterized in transgenic mice with overexpression of functional human 5-HT_4_ receptors selectively in the heart (5-HT_4_-TG, e.g., Gergs et al. 2010, 2013). For instance, we have used 5-HT_4_-TG to detect cardiac effects on 5-HT_4_-serotonin receptor agonists like prucalopride, cisapride, and metoclopramide (Keller et al. 2018, Neumann et al. 2021a). In parallel, we have then studied human atrial preparations (HAP): HAP were investigated to test the clinical relevance of our studies in 5-HT_4_-TG (Keller et al. 2018, Neumann et al. 2021a).

As a starting point for the present study, we noticed ancient experiments in the gut: others reported, four decades ago, that positive contractile effects of serotonin in isolated guinea-pig ileal preparations were antagonized by bromopride (Fontaine and Reuse 1978). At that time, this was not understood on a receptor level. We now know that these contractions in the gut of the guinea pig are 5-HT_4_-serotonin receptor mediated (Waikar et al. 2012). Thus, their contractile data (Fontaine and Reuse 1978) are consistent with an antagonistic effect of bromopride on gut 5-HT_4_ serotonin receptors (Waikar et al. 2012).

To the best of our knowledge, the action of bromopride on 5-HT_4_-serotonin receptors in the heart of experimental animals or on isolated preparations from the human atrium has never been reported before.

In summary, we tested the following hypotheses of increasing clinical relevance:

1. Bromopride stimulates the FOC in isolated left atrial preparations (LA) from 5-HT_4_-TG.
2. Bromopride augments the beating rate in spontaneously beating right atrial preparations (RA) from 5-HT_4_-TG.
3. Bromopride raises FOC in HAP via 5-HT_4_-serotonin receptors.

## Materials and methods

### Transgenic mice

#### Contractile studies in mice

In brief, LA or RA from 5-HT4-TG and their litter mates (random gender, about 150 days of age) were isolated and mounted in organ baths as previously described (Gergs et al. 2010, 2013; Neumann et al. 2021b). The bathing solution of the organ baths contained (in mM) 119.8 NaCI, 5.4 KCI, 1.8 CaCl_2_, 1.05 MgCl_2_, 0.42 NaH_2_PO_4_, 22.6 NaHCO_3_, 0.05 Na_2_EDTA, 0.28 ascorbic acid, and 5.05 mM glucose. Ascorbic acid is used in this buffer in order to impair oxidation and thence inactivation of drugs like serotonin. The solution was continuously gassed with 95% O_2_ and 5% CO_2_ and maintained at 37 °C and pH 7.4 (Neumann et al. 1998). Spontaneously, RA from mice were used to study any chronotropic effects. In LA from mice, samples were stimulated with square wave forms for 5-ms duration with a voltage 10% higher above threshold to initiate contractions. Under isometric conditions, a bridge amplifier fed signals into a commercial personal computer. The drug application was as follows. After equilibration was reached, bromopride and where indicated in legends also GR125487 (a 5-HT4 serotonin receptor antagonist: Schiavi et al. 1994) were added to LA or RA preparations. In separate experiments, we first gave 1 µM serotonin and then we added 10 µM bromopride.

#### Contractile studies on human preparations

The contractile studies on HAP were done using the same setup, stimulation conditions, and buffer as used in the mouse studies in the preceding paragraph. The samples were obtained from male patients and female patients, aged more than 50 years. Morbidities included severe coronary heart disease, hypertension, heart failure, and atrial fibrillation. Drug therapy comprised acetyl salicylic acid apixaban, hydrochlorothiazide, furosemide, and bisoprolol. Our methods used for atrial contraction studies in human samples have been previously published and were not altered in this study (Gergs et al. 2009, 2017). In principle, patients underwent bypass surgery and we obtained muscle strips from the right appendage. Tissue was rapidly transferred from the theater to the laboratory. Patients gave written informed consent to participate in this study.

Drug application was like in the preceding paragraph. In addition, we also gave bromopride in the presence of cilostamide (a phosphodiesterase III-inhibitor). Phosphodiesterase III is the main isoform in the human heart (e.g., Molenaar et al. 2013, Christ et al. 2006). We used low concentrations of cilostamide that barely increased FOC, in order not to overlook a PIE of a subsequently applied drug. We and others used this procedure successfully in many previous studies in HAP (e.g., Christ et al. 2006; Gergs et al. 2023).

### Data analysis

Data shown are means ± standard error of the mean. Statistical significance was estimated using the analysis of variance followed by Bonferroni’s *t*-test paired or unpaired Student’s *t*-test as appropriate and explained in the figure legends. A *p*-value < 0.05 was considered to be significant.

### Drugs and materials

The drugs isoprenaline-hydrochloride, bromopride (10 mM was dissolved in dimethylsulfoxide (DMSO)), serotonin, and 5-fluoro-2-methoxy-[1-[2-[(methylsulfonyl)amino]ethyl]−4-piperidinyl]−1*H*-indole-3-methylcarboxylate sulfamate (GR125487, Ki of 0.19 nM: Schiavi et al. 1994) were purchased from Sigma-Aldrich (now Merck, Dreieich, Germany) or MedChemExpress via Hycultec (Beutelsbach, Germany). All other chemicals were of the highest purity grade commercially available. Deionized water was used throughout the experiments. Stock solutions were prepared fresh daily.

## Results

**Fig. 3:**
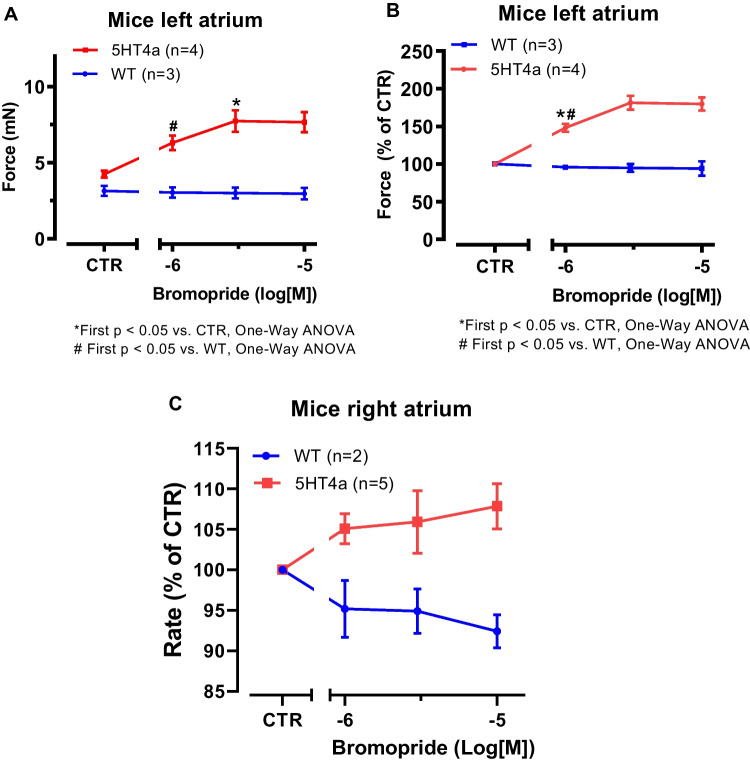
**A** Summarized data for the concentration-dependent effect of cumulatively applied bromopride on force of contraction in left atrial preparations from 5-HT_4_-TG (squares) and WT (circles) for comparison. Ordinates plot force of contraction in millinewton (**A**) and in percentage of pre-drug value (CTR, **B**) or beating rate (**C**). Abscissae indicate concentrations of bromopride in decadic logarithm of molar concentrations. Significant differences are indicated with an asterisk and a number sign. Numbers in brackets mean number of experiments

Bromopride, when given alone, exerted a concentration-dependent PIE in LA from 5-HT_4_-TG. This is seen in millinewton (Fig. 3A) and in percentage of pre-drug value (Fig. 3B). In contrast, bromopride failed to raise FOC in LA from WT (Fig. 3A and B). If bromopride behaved like 5-HT, bromopride should exert effects on the beating rate in RA of 5-HT_4_-TG. Indeed, we noticed a concentration-dependent positive chronotropic effect of bromopride that is plotted in Fig. 3C.

**Fig. 4:**
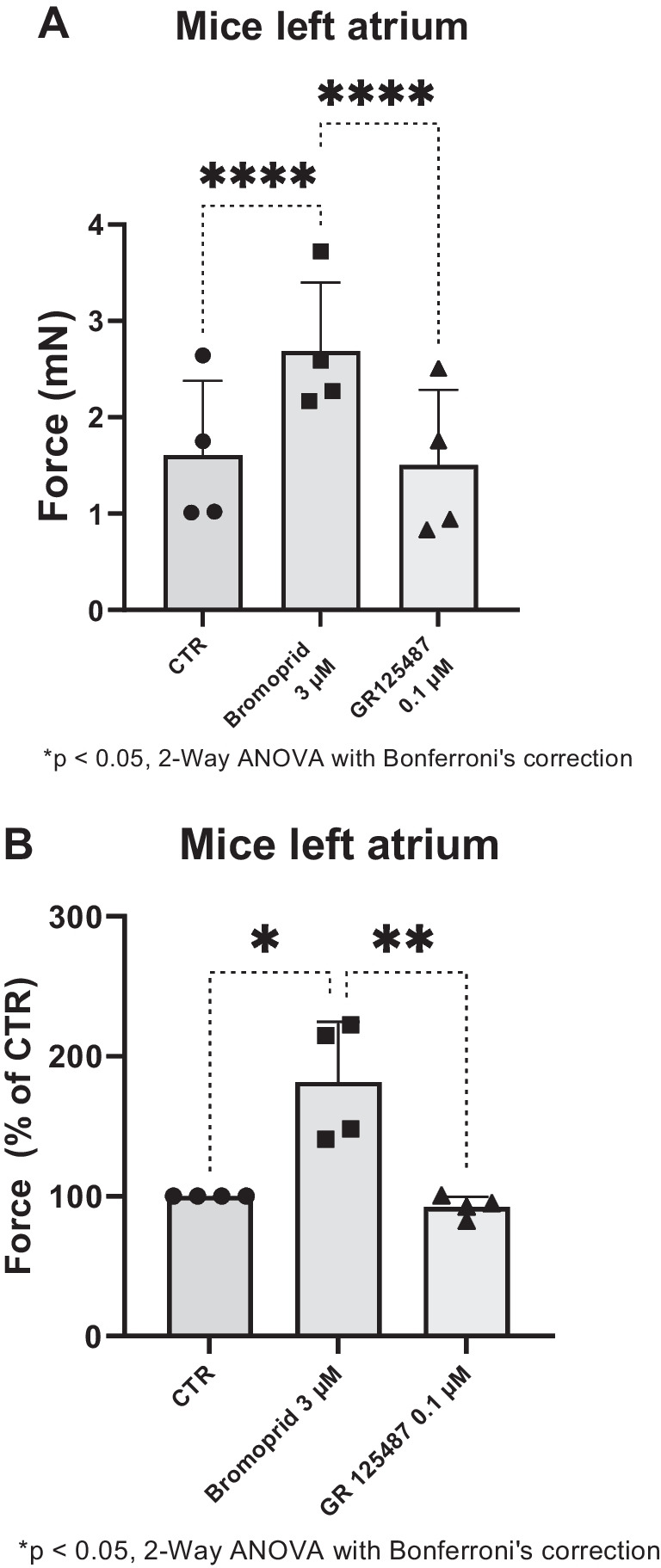
**A** Summarized data for the effect of bromopride alone or in the presence of GR125487 on force of contraction in mN or % of pre-drug value (**B**) in left atrial preparations from 5-HT_4_-TG. Significant differences are indicated with an asterisk. Points in bars mean number of experiments

To corroborate the view that bromopride acts via 5-HT_4_-receptors, we depict that GR125487, an antagonist at 5-HT_4_-serotonin receptors (Gergs et al. 2010, 2013; Schiavi et al. 1994), reversed the positive inotropic effect of bromopride in LA of 5-HT_4_-TG. Several such experiments are summarized in regard to FOC measured in millinewton (Fig. 4A) and in percentage (Fig. 4B).

**Fig. 5:**
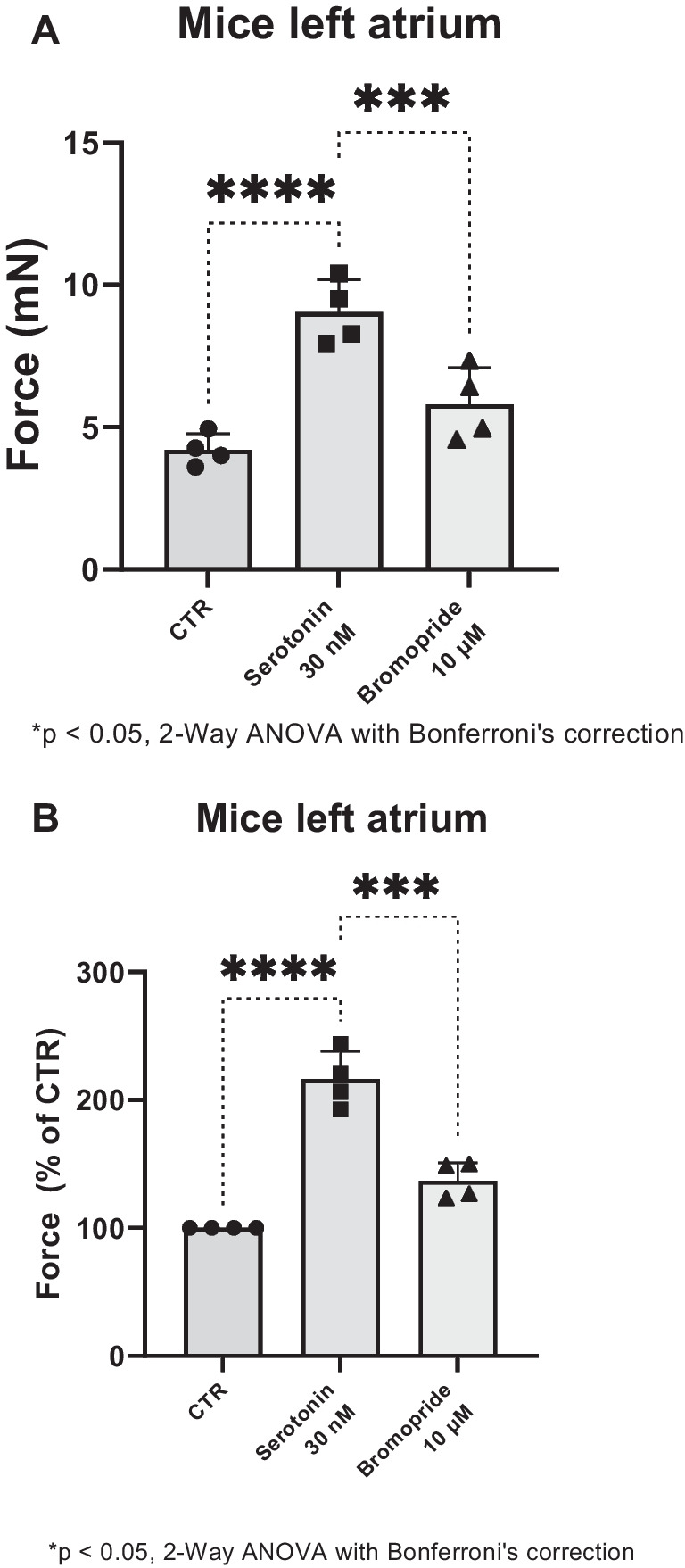
**A** Summarized data for the effect of serotonin alone or in the presence of bromopride on force of contraction in mN or % of pre-drug value (**B**) in left atrial preparations from 5-HT_4_-TG. Significant differences are indicated with an asterisk. Points in bars mean number of experiments

In a different set of experiments, we first increased FOC by serotonin. Additionally applied bromopride exerted a concentration- and time-dependent negative inotropic effect in LA from 5-HT_4_-TG (Fig. 5A and B). These findings can be interpreted as evidence that bromopride acts as a partial agonist in LA of 5-HT_4_-TG.

**Fig. 6:**
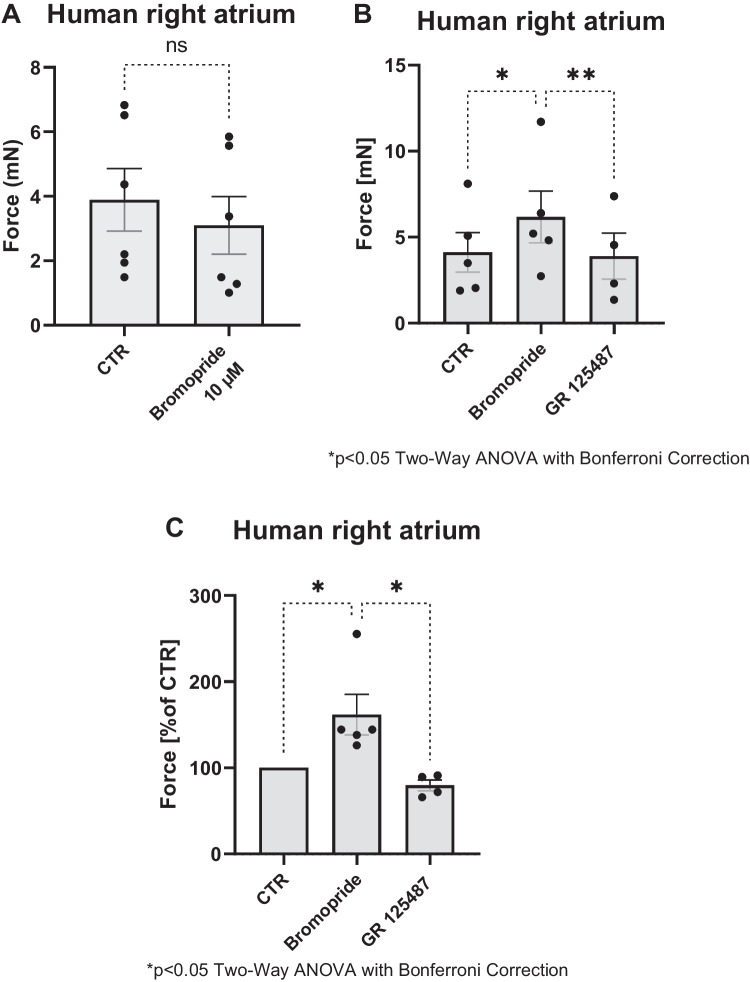
**A** Summarized data for the effect of bromopride (10 µM) alone, without the presence of cilostamide. In contrast, **B** and **C** show the effect of bromopride (10 µM) with 100 nM cilostamide or in the additional presence of GR125487 (1 µM) on the force of contraction, measured in mN (**B**) or in % of pre-drug value (**C**) in human right atrial preparations. Significant differences are indicated with an asterisk. The points in bars represent the number of experiments

Of course, it was more clinically relevant to study bromopride based on this animal studies in the human heart. As a tool to this end, we stimulated HAP electrically, added bromopride, and thus studied the PIE of bromopride. Bromopride alone was ineffective to raise FOC in HAP (Fig. 6A). Bromopride was effective to augment FOC in HAP when FOC in preparations was first elevated by a single low concentration of cilostamide (100 nM) and then bromopride was added (Fig. 6B and C). We have used low-cilostamide concentrations repeatedly to unveil PIE of 5-HT_4_ serotonin receptors agonists (Gergs et al. 2023; Neumann et al. 2024a, b, c, d; Hesse et al. 2024). GR125847 reduced the PIE of bromopride in HAP (Fig. 6B and C).

**Fig. 7:**
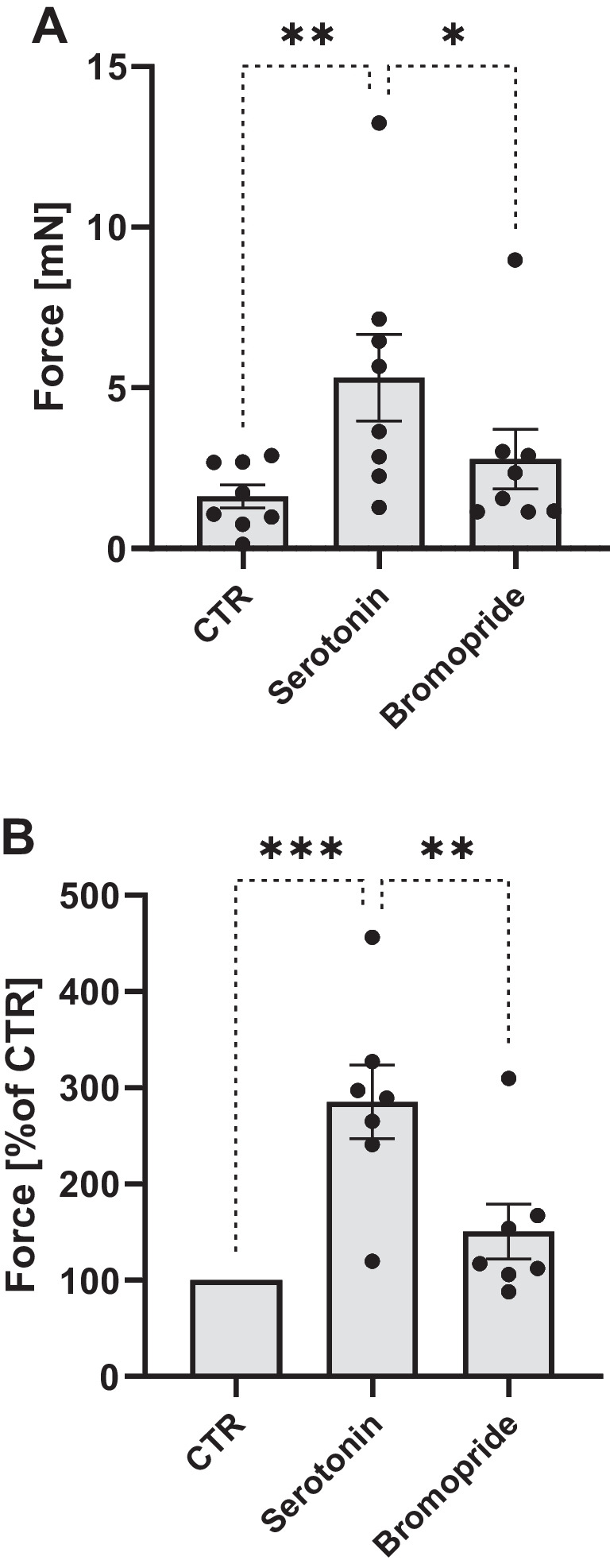
**A** Summarized data for the effect of serotonin (1 µM) alone or in the presence of bromopride (10 µM) on force of contraction in mN or % of pre-drug value (**B**) in human right atrial preparations. Significant differences are indicated with an asterisk. Points in bars mean number of experiments

Moreover, one can ask why bromopride itself failed to raise FOC (Fig. 6A). A partial agonism might explain this. Hence, we designed the following experiments: First, we stimulated HAP with 1 µM serotonin. Thereafter, a single concentration of 10 µM bromopride was added. We noted that under these conditions bromopride reduced FOC in HAP. This is quantified with respect to FOC in several experiments (Fig. 7A and B). Hence, as in mice, bromopride acted as a partial agonist at 5-HT_4_-serotonin receptors in HAP.

## Discussion

### Cardiac mechanism(s) of bromopride

We suggest that bromopride increased force of contraction and beating rate as an agonist at cardiac human 5-HT_4_-receptors because bromopride only increased contractility in atrium from 5-HT_4_-TG and not in WT. Moreover, the PIE of bromopride in LA and HAP was antagonized by an antagonist at 5-HT_4_-serotonin receptors (GR125487, Schiavi et al. 1994).

This pharmacology of bromopride is in line with our previous work on other chemically similar drugs that share a benzamide structure. Similar to 5-HT and some other agonists like metoclopramide, mosapride, zacopride, or cisapride, bromopride increased FOC in HAP only in the presence of cilostamide (Neumann et al. 2024a, b, c, d). This can be explained if we assume that bromopride acts via the same signal transduction pathways after receptor stimulation as serotonin: the cAMP is augmented, the cAMP-dependent protein kinase raises the phosphorylation state of phospholamban, more Ca^2+^ enters the sarcoplasmic reticulum per unit of time, and relaxation is speedier. Now more and faster Ca^2+^ can be released from the sarcoplasmic reticulum and rate of contraction and FOC climbed (Gergs et al. 2010). This would adequately explain the contractile effects of bromopride in HAP and LA from 5-HT_4_-TG. At least after intraperitoneal injection of bromopride in rats, the hypothalamic activity of adenylyl cyclase was enhanced (Portaleone et al. 1978). This was conceivably so because bromopride entered the hypothalamus and stimulated 5-HT_4_ receptors, which were at that time unknown; and hence, no antagonists were employed (Portaleone et al. 1978). However, these previous findings make it likely that a similar mechanism is operational in the human heart.

Another argument for this signaling pathway (Fig. 1B) can be made: the PIE of bromopride is only measurable in the presence of cilostamide, a phosphodiesterase III inhibitor, in HAP. This finding is consistent with the view that bromopride via adenylyl cyclase raised cAMP. This cAMP was degraded by phosphodiesterase III and therefore inactive. When we inhibited this degradation of cAMP with cilostamide, we noticed a PIE to bromopride, suggesting that bromopride acts via cAMP (Fig. 1B). In HAP, stimulation of cAMP by bromopride is apparently too low to lead to an increase in FOC. But when degradation of cAMP is impaired by cilostamide then force is boosted by bromopride. This hypothesis is in line with recent reports on other prokinetic drugs or anti-Alzheimer drugs from our group (mosapride: Neumann et al. 2024a, zacopride: Neumann et al. 2024b, MR33317: Neumann et al. 2024c, tegaserod: Hesse et al. 2024). Likewise, using psilocin or dimethyltryptamine, both hallucinogenic drugs, we only noted a PIE via 5-HT_4_ receptors in HAP when we impaired the degradation of cAMP in HAP with cilostamide (Dietrich et al. 2023; Neumann et al. 2024d).

Theoretically, bromopride may also act as an antagonist at D_2_-dopamine receptors in the heart or as a blocker of sodium channels in the heart, like its mother compound procainamide, a class I antiarrhythmic drug according to the classification of Vaughan Williams (Fig. 1B). However, D_2_-dopamine receptors are probably irrelevant in the human heart for the regulation of FOC (review: Neumann et al. 2023b). Hence, they cannot explain why bromopride increased FOC in HAP. Moreover, inhibition of sodium ion channels usually decreases FOC; and therefore this inhibition cannot explain a PIE of bromopride.

In addition, bromopride, like its sister compound metoclopramide (Kuchel et al. 1985), might conceivably release catecholamines and thus might indirectly elevate FOC. However, bromopride led to a rise in FOC only in LA from 5-HT_4_-TG and not in LA from WT, where bromopride should also release noradrenaline if that is a relevant mechanism for the PIE of bromopride. Moreover, the PIE in LA of 5-HT_4_-TG and in HAP was antagonized by 5-HT_4_ receptor antagonists which is not an antagonist at β-adrenoceptors and thence released noradrenaline in the heart and is unlikely to play a role in the PIE of bromopride.

### Species and drug differences

Of note, bromopride alone climbed FOC in LA from 5-HT_4_-TG and not in HAP. Only in the presence of cilostamide (Fig. 1B), bromopride led to a growth in the FOC in HAP. This is in line with our previous work on other 5-HT_4_-agonists like LSD (Gergs et al. 2023). In contrast, metoclopramide alone that is in the absence of cilostamide enlarged FOC in HAP (Neumann et al. 2021a). We would therefore speculate that the intrinsic affinity at the 5-HT_4_ receptor of bromopride is smaller than that of metoclopramide in HAP. Apparently, changing a bulky bromine atom to a chlorine atom greatly alters the ligand receptor affinity in our contraction studies in HAP (Fig. 1B). But this is speculative. Moreover, it needs to be explained why bromopride alone increased FOC in LA from 5-HT4-TG but not in HAP. We speculate that this is due to the higher expressional levels of 5-HT4-receptors in 5-HT4-TG than in the human atrium (Kaumann et al. 1996, Neumann et al. 2021b). We do not think that the bromopride is more potent in LA from 5-HT4-TG because of a more prominent role of PDE IV in the mouse heart and a more prominent role of the PDE III in the human heart. At least we know that the efficacy of serotonin to raise FOC in LA of 5-HT4-TG is not enhanced by cilostamide but only by rolipram (a PDE IV inhibitor: Neumann et al. 2019).

### Regional differences

Serotonin increases FOC in the left and right atrium of all patients studied (Kaumann et al. 1990, Kaumann and Levy 2006). However, in human ventricular tissue, no PIE to serotonin was noted (Jahnel et al. 1992). This was later confirmed. It turned out that in ventricular muscle strips from patients with non-failing ventricles (= no overt heart failure clinically) and non-failing rat ventricular muscle preparations, serotonin had no PIE (Brattelid et al. 2004, Brattelid et al. 2007). This was correlated and conceivably caused by a lower expression of the receptor (as mRNA) in the non-failing human heart ventricle compared to the human atrium (Brattelid et al. 2004). However, when patients suffered from heart failure (or when heart failure was experimentally induced in rat ventricle) then there was a PIE and then the receptor number was found to be increased (Brattelid et al. 2004, Brattelid et al. 2007). Hence, current thinking is that serotonin is important in all patients in the atrium but only in heart failure in the ventricle (discussed in Neumann et al. 2023a).

### Clinical relevance

Peak therapeutic plasma levels of bromopride in patients have been reported around 0.1–1 µM, well in the range where we noted a start of contractile effects of bromopride (Dunne et al. 2013). Some studies used intravenously injected bromopride (Epifanio et al. 2018). In such cases, rather high peak plasma concentrations of bromopride would occur. In intoxications, much higher plasma levels of bromopride are expected leading to more effects in the human heart. Moreover, bromopride is metabolized in the liver in humans (Dunne et al. 2013). It is chemically probably metabolized by cytochromes because oxidations of bromopride occurred (Dunne et al. 2013). However, the metabolism of bromopride and the role of isoforms of cytochromes are understudied. Nevertheless, it is well accepted that many drugs can inhibit such liver cytochromes. The inhibition of the enzymatic activity of these cytochromes (by drugs or due to genetic defects: slow metabolizers) would reduce the metabolism of bromopride and this would further augment plasma concentrations.

### Limitations of the study

One can argue that we have not tested the effects of bromopride on the sinus node of man directly. Such a study would require access to the human pacemaker. Such studies were beyond the scope of this initial study. Furthermore, we did not have the opportunity to study contractility in insolated human ventricular muscle strips for lack of access to that tissue. However, 5-HT_4_ receptors are not functionally active in the non-diseased human ventricle (Brattelid et al. 2004, Qvigstad et al. 2005). Bromopride is not expected to be used in patients with heart failure and thence ventricular effects of bromopride are unlikely to pose a clinical problem, at least for the healthy human heart (Qvigstad et al. 2005). The concentrations of serotonin in the human heart have been measured as 0.25 to 2.3 µM serotonin (Sole et al. 1979). Hence, based on these data, the 5-HT_4_-antagonistic effects of bromopride might occur in the living human heart. Moreover, the plasma serotonin levels are higher in heart failure patients than in control patients (Selim et al. 2017, Neumann et al. 2023a, b). We noted a PIE to bromopride only when we inhibited PDE III by cilostamide. This might occur in some cases in the clinic. Levosimendan and pimobendan are clinically used drugs and are mainly PDE III inhibitors (von der Leyen et al. 1991; Orstavik et al. 2014; Rayo et al. 2023).

In summary, we can now address the hypotheses raised in the “Introduction” in this way:

1. Bromopride stimulates the FOC in isolated left atrial preparations from 5-HT_4_-TG.
2. Bromopride augments the beating rate in spontaneously beating right atrial preparations from 5-HT_4_-TG.
3. Bromopride acts as a partial agonist via 5-HT_4_-serotonin receptors in isolated human atrial preparations.

## Data Availability

All source data for this work (or generated in this study) are available upon reasonable request.
